# Prevalence of the Gingival Phenotype in Adults and Associated Risk Factors: A Systematic Review of the Literature

**DOI:** 10.3390/clinpract14030064

**Published:** 2024-05-08

**Authors:** Sophie-Myriam Dridi, Clément Ameline, Jean Michel Heurtebise, Séverine Vincent-Bugnas, Carole Charavet

**Affiliations:** 1 Département de Parodontologie, Faculté de Chirurgie Dentaire, Université Côte d’Azur, 06800 Nice, France; cleameline@gmail.com (C.A.); vincent-bugnas.s@chu-nice.fr (S.V.-B.); 2 Unité de Parodontologie, Pôle Odontologie, Centre Hospitalier Universitaire de Nice, 06800 Nice, France; jeanmiheu@gmail.com; 3 Laboratoire MICORALIS, Université Côte d’Azur, UPR 7354, 06800 Nice, France; c.charavet@gmail.com; 4 Département d’Orthodontie, Faculté de Chirurgie Dentaire, Université Côte d’Azur, 06800 Nice, France; 5 Unité d’Orthodontie, Pôle Odontologie, Centre Hospitalier Universitaire de Nice, 06800 Nice, France

**Keywords:** gingival phenotype, gingival biotype, gingival morphotype, prevalence

## Abstract

The relevance of assessing the gingival phenotype prior to the initiation of periodontal, orthodontic, or prosthetic therapy has been clearly demonstrated. However, publications on this subject are either old or concerned with the means of assessing the gingival phenotype or the main factors likely to modify it. The main objective of this systematic review of the literature was therefore to investigate the prevalence of different gingival phenotypes in adults in good general health and with a healthy periodontium. A systematic review of the literature was performed following the guidelines of PRISMA recommendations using an electronic search strategy on four databases (PubMed, Scopus, Cochrane Library, and Embase) complemented by a manual search. Three independent authors were involved in study selection, data extraction, and bias assessment. Results: Of 807 articles, 17 of them, published between 2012 and 2023, involving 3277 subjects from 11 countries and 9766 dental sites, fulfilled the inclusion criteria. The prevalence of the gingival phenotype could not be determined at the level of an individual or a dental arch because all the publications assessed this phenotype only at the level of certain dental sectors, and were not chosen at random. The maxillary central incisors and maxillary or mandibular first molar sectors were associated with a high and thick gingival phenotype, independently of the dental morphology, gender, and age of adult subjects. Furthermore, in these regions, this gingival phenotype tended to be associated with a thick vestibular bone table. In contrast, maxillary and mandibular incisors and premolars more often had a thin gingival phenotype. For other teeth, the results were less conclusive. It is important not to rely solely on the overall appearance of the dentition but to independently assess the thickness and height of the gingiva at each dental site requiring intervention. Finally, this study highlights a key point, namely the need for further longitudinal studies to determine the prevalence in healthy adults. For practicality and feasibility reasons, these studies should be designed according to therapeutic needs, dental sector by dental sector, and within homogeneous source populations. PROSPERO registration: CRD 42023392602.

## 1. Introduction

The gingival phenotype, otherwise known as the gingival biotype or morphotype, defines all the morphological characteristics of the superficial periodontium at a given moment [1]. For each individual, it is the result of both innate and acquired factors. In general populations, several types can be diagnosed, depending on whether the gingiva is thin or thick, high, or reduced [2,3]. In periodontics, their evaluation is an essential clinical criterion to determine whether periodontal therapies are preventive or curative and non-surgical or surgical [4,5,6]. Many authors have shown, for example, that a thin gingival phenotype, as opposed to a thick gingival phenotype, is more often associated with the risk of gingival recession of infectious or traumatic origin, including after periodontal surgery, orthodontic treatment, extraction, or the fitting of a prosthesis fixed at the intrasulcular margins [4,6,7,8,9,10]. Furthermore, a fine gingival phenotype is thought to reduce the prognosis of periodontal plastic surgery [5].

Thus, in daily practice, an assessment of the gingival phenotype is relevant because it enables clinicians to predict the risk of formation or worsening of gingival substance loss, personalize preventive and curative periodontal treatments, and evaluate their results over time.

However, to our knowledge, there is no recent systematic review of the literature that specifies the prevalence of different gingival phenotypes in adults in good general health with a healthy periodontium. Indeed, the most recent reviews devoted to this subject were either published several years ago [2] or focused on the means of assessing the gingival phenotype or on the main risk indicators/factors likely to modify it [3,11,12,13].

The primary objective of this systematic literature review was to investigate the current prevalence of gingival phenotypes in generally healthy individuals with a healthy periodontium. The secondary objective was to assess the impact of intrinsic and extrinsic risk indicators/factors on the nature of the gingival phenotype, with the aim of better detecting the most fragile phenotypes or those that could be weakened by a dental procedure and of personalizing periodontal therapy.

## 2. Materials and Methods

### 2.1. Recording the Protocol

This review was carried out in accordance with the PRISMA (Preferred Reporting Items of Systematic Reviews and Meta-Analysis) recommendations updated in 2021 [14]. The PRISMA checklist is provided as Supplementary Material. This study was registered in the international PROSPERO database (Prospective Register of Systematic Review) under the number CRD 42023392602.

### 2.2. Research Question and Eligibility Criteria

In order to meet the objectives of this literature review, the research question was formulated as follows: what are the prevalence and associated risk factors of gingival phenotypes in adults?

To determine the inclusion and exclusion criteria for the studies, we used the acronym PICOS (Population, Intervention, Comparison, Outcome, Study design):

(P) Population: adults aged at least 18 years, regardless of gender, in good health (free of general disease or controlled general disease) and with a healthy periodontium.

(I) Procedure: measurement of the height and thickness of the gingiva, regardless of the method of assessment, on one or more permanent natural teeth.

(C) Control: no comparison or comparison according to an individual variable.

(O) Primary endpoint: assessment of the prevalence of the gingival phenotype.

(S) Study design: retrospective, cross-sectional, or prospective study evaluating the quantity and quality of the gingival phenotype.

The inclusion criteria were:

i. A retrospective, cross-sectional, or prospective study assessing the quantity and quality of the gingival phenotype.
ii. At the level of one or more dental sectors.
iii. Including adult subjects in good general health, regardless of gender, with a healthy periodontium.
iv. The articles must be written in English or French and published between 2011 (1 January 2011) and 29 January 2024.

Exclusion criteria included interventional studies on periodontal surgery or peri-implant tissues; case reports, systematic reviews, and meta-analyses; publications based on previously published cohorts; in vitro, ex vivo, and animal studies; authors’ opinions; expert opinions; questionnaires; and editorials.

### 2.3. Search Strategy and Equations

This systematic review was carried out using four electronic bibliographic databases: PubMed (Medline), Cochrane Library, Embase (Experta Medical Database by Elsevier), and Scopus (Elsevier). The search equations, including Boolean operators, are detailed in Table 1.

In addition, to complete the electronic search, a manual search was carried out using the bibliography of the selected articles and the search engines of five specialist dental journals indexed in PubMed (Journal of Clinical Periodontology, Journal of Periodontology, Journal of Periodontal Research, Journal of Dental Research, and Journal of Dentistry).

The search for articles was completed on 29 January 2024.

### 2.4. Selection of Articles

The two principal investigators (CA, SMD) independently assessed the eligibility of the articles according to a standardized protocol. In the event of a disagreement that could not be resolved through discussion, the intervention of a third investigator (CC) was requested as to whether to make a final decision on the selection of studies deemed contentious.

The selection protocol was based on the following steps: after eliminating duplicates among databases using bibliographic reference management software (Zotero 5.0.96.3), articles were selected on the basis of their title, then their abstract, and finally their full reading.

This protocol made it possible to draw up four successive lists of articles:

− List 0 included all the articles obtained from the search equations.
− List no. 1 included all the articles selected from list 0 after eliminating duplicates and reading the titles.
− List no. 2 included all the articles selected from list no. 1 after reading the abstracts and then the full texts.
− List no. 3 finally included all the articles from list no. 2 and the manual search after a full reading of the additional articles deemed relevant.

#### Data Extraction and Risk of Bias Assessment

Data collection and synthesis were carried out independently and in a standardized manner by the two main reviewers (CA, SMD) based on articles that met the inclusion criteria. For each article, the following information was recorded on data extraction sheets, predetermined by all the authors:

− Author, journal, and year of publication; type of study.
− Objective(s).
− Population (number of subjects, individual characteristics, teeth concerned).
− Inclusion and exclusion criteria.
− Materials and methods (intervention, protocol).
− Results (prevalence, association).
− Selection, confusion, and classification bias.

The search for bias was blinded by the same reviewers as before, using the Mixed Methods Appraisal Tool (MMAT) [15]. This tool allows for the qualitative assessment of several types of studies via five major questions and three answer possibilities: yes, no, do not know. For each study, a final score is obtained as follows: 20% is awarded for each yes answer and 0% for the other two answers. The maximum score is 100%, indicating a very low risk of bias. The minimum score is 0% and corresponds to a very high risk of bias. In order to harmonize the interpretation of the questions, the investigators agreed beforehand on the nature of the evaluation criteria for the main biases.

### 2.5. Statistical Analysis

Given the differences in the quality of the internal validity of the studies, it was not possible to conduct a meta-analysis. Consequently, the results presented are descriptive only. Only inter-examiner agreement was assessed using Cohen’s kappa test.

## 3. Results

### 3.1. Article Selection

Our search strategy produced an initial list of 807 articles from the search engines used. Nine duplicates were removed, and 752 articles were not retained based on their title. Of the 46 articles in list no. 1, a further selection, based on reading the abstracts, resulted in the exclusion of 32. Finally, of the 14 articles eligible for full reading, only 13 were included [16,17,18,19,20,21,22,23,24,25,26,27,28]. The article by Das et al. (2022) was not retained due to the lack of precision of the gingival thickness endpoint [29].

Regarding the final eligibility of the articles, the agreement between the lead reviewers was described as perfect (no disputes).

In addition, the manual search was used to select four other studies [30,31,32,33]. List no. 3 consisted of 17 articles that met the inclusion criteria.

**Figure 1:**
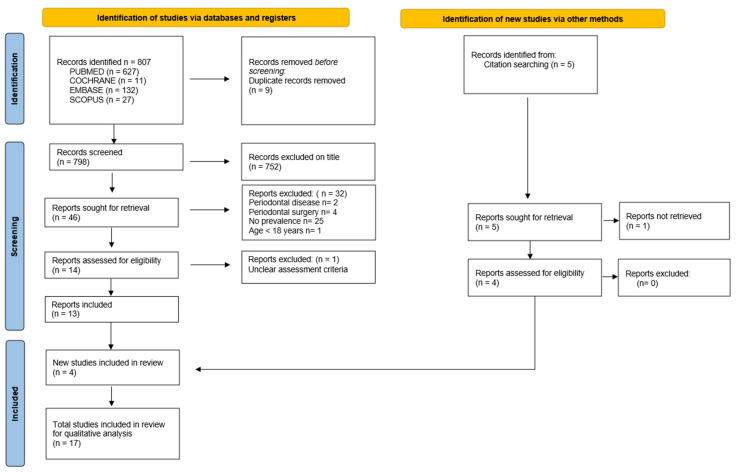
Flowchart of the research process according to the PRISMA 2021 recommendations (Page et al. 2021) [14].

Details of the study selection process are shown in Figure 1.

### 3.2. Study Characteristics

#### 3.2.1. General Characteristics

- Type of study.

All the studies included are cross-sectional, observational studies published between 2012 and 2023. The characteristics of each study are described in Table 2.

Except for the Shetty and Bhat (2022) study [17], all authors mentioned approval by an institutional ethics or medical committee.

- Geographical location of populations.

Four studies were conducted in India [17,18,22,32], two in China [31,33], one in Singapore [23], one in Yemen [24], one in Saudi Arabia [16], three in Germany [21,26,30], one in Portugal [19], one in the Dominican Republic [25], one in the USA [20], one in Brazil [27], and one in Malaysia [28].

Ten studies came from Asia, four from Europe, and three from America. The gingival phenotype was not studied for populations in Africa and Oceania. In Europe in particular, three studies were carried out in one northern country and one study in a southern country.

- Characteristics of subjects.

The number of subjects included per study ranged from 800 [32] to 31 [31], and for all studies, it totaled 3277, with 1552 women and 1362 men, at least. We could not indicate exactly the overall distribution of subjects by gender, as Singh et al. (2016) did not take this individual criterion into account [22].

All the individuals included in the 17 studies in the review were healthy adults, free of general pathology. In 15 studies, the authors also mentioned that none of the subjects were taking medication with known effects on the superficial periodontium [16,19,20,21,22,23,24,25,26,27,28,30,31,32,33]. In addition, only three studies mentioned smoking as a non-inclusion criterion [18,27,31]. For Fischer et al. (2014, 2015, 2022), the tobacco consumption of the subjects selected was not precisely determined, but overall, it should not exceed 10 cigarettes/day [21,26,30].

The distribution of women and men was perfectly balanced in three studies [16,18,32], relatively well balanced in favor of women in seven studies [20,21,23,24,30,31,33], clearly in favor of women in four studies with a rate ≥ 60% [19,26,27,28], and clearly in favor of men in two studies with a rate ≥ 60% [17,25]. The team of Singh et al. (2016) did not specify the gender distribution [22].

In 13 studies, the subjects were young, with an average age between 22 and 32 years [16,18,21,23,24,25,26,27,28,30,31,32,33]. In the Frost et al. (2015) study [20], the average age was higher, around 53 years, and in Singh et al. (2016) study [22], the average age was not specified; the authors only mentioned that the individuals included were young and aged 18 years or more. Shetty and Bhat (2013) did not specify the average age of their source population, either, but they did mention two groups, one formed by individuals aged 18–30 and the other by individuals aged 30–50 [17]. The study by Peixoto et al. (2015) did not indicate the age of the subjects [19].

#### 3.2.2. Protocol and Assessment Criteria

- Selection of subjects.

Only three teams determined how to calculate the number of subjects required [24,27,28].

Furthermore, the source populations were heterogeneous. In eight studies, the subjects were dental or stomatology students, selected from their respective academic focus [21,22,26,27,28,30,31,33]. In six studies, patients were included during or after a first scheduled consultation at a dental facility [16,17,20,23,24,32]. In one study, patients were recruited voluntarily at their place of residence and then examined in a dental establishment [25], and in two studies, no information was given on the organization of recruitment [18,30].

Of the abovementioned studies, only two teams specified a random recruitment method without confirming the existence of a systematic consecutive method [24,25].

- Choice of dental sectors.

Regarding the dental sector analyzed, most authors focused on the maxillary incisivo–canin sector [16,17,18,19,21,22,25,27,28,30,32,33]. Other teams chose to integrate the mandibular incisivo–canin sector [31] and/or the maxillary or mandibular premolars and first molar [20,23,24,26].

In total, the gingival phenotype was assessed in 9766 teeth: 7939 maxillary and 1827 mandibular. In all the studies, the gingival phenotype was assessed much less often in mandibular teeth (18.7% of all the teeth examined).

More specifically, the authors assessed the gingival phenotype.

—In the maxilla: 3397 central incisors, 1826 lateral incisors, 1370 canines, 200 first premolars, 92 second premolars, and 1054 first molars (respectively, 42.78%, 22.96%, 13.23%, 2.51%, 1.15%, and 13.27% of the maxillary teeth examined). Thus, the gingival phenotype of the central incisors was by far the most studied.

—In the mandible: 217 central incisors, 162 lateral incisors, 162 canines, 141 first premolars, 94 second premolars, and 1051 first molars (respectively, 11.87%, 8.80%, 8.80%, 7.71%, 5.11%, and 57.52% of the mandibular teeth examined). The gingival phenotype of the first molars was largely represented.

For all the teeth examined, the gingival phenotype of the maxillary central incisors was studied the most (34.8%), followed by that of the maxillary lateral incisors (18.7%) and the maxillary first molars (10.1%). Conversely, the gingival phenotype of the maxillary and mandibular second premolars was evaluated the least (less than 1% for each type of tooth).

- Periodontal health assessment criterion.

The periodontal health endpoint was precisely defined only in the studies by Lee et al. (2018) [23] (inclusion of healthy intact or reduced periodontal with a Full-Mouth Bleeding Score ≤ 15%) and Shao et al. (2018) [31] (gingival index < 1, probing depth < 3 mm, no clinical attachment loss, no radiographic alveolysis).

In six studies, the authors only indicated the performance of a periodontal sanitation session several days prior to clinical data collection [21,24,26,30,31,32], and in the others, no details were mentioned on the actual state of the gingival [16,17,18,19,20,22,25,27,28,33]. The latter authors only cited the existence of healthy gingiva as an inclusion criterion and the presence of specific clinical signs of periodontitis as an exclusion criterion.

- Gingival phenotype assessment criterion.

The majority of authors assessed marginal gingival thickness via the periodontal probe transparency test. The type of probe varied among studies and was not determined by Shetty et Bhat (2013) [17]. Nine teams used the standardized PCP UNC-15 (Hu-Friedy^®^, Chicago, IL, USA) metal probe recommended by the American Academy of Periodontology and the European Federation of Periodontology [20,21,22,23,25,27,28,30,32]. Three teams used the Williams (Hu-Friedy^®^, Chicago, Il, USA) metal probe [17,32,34], one team the OMS (Henry Schein^®^, USA) metal probe [19], and one team the yellow-colored (Deppeler SA^®^, Rolle, Switzerland) plastic PCC 12 probe [26]. Nik-Azis et al. (2023) [28] used the plastic Colorvue Biotype Probe or CBP probes colored blue, green, and white at the tips (Hu-Friedy^®^, Chicago, IL, USA).

Direct measurement of gingival thickness was also estimated by transfixing an endodontic file within the attached gingival tissue in five studies [18,20,23,24,31], via caliper for the Fischer et al. (2015) study [20] and via CBCT (Cone Beam Computer Tomography) imaging in the Shao et al. (2018) study [31].

Seven teams used two or three means of assessment [18,20,23,24,27,28,31].

The reference values for gingival thickness, which enabled the authors to classify the gingiva as thick or thin, were justified and precisely indicated in all the studies.

For the periodontal probe transparency test, 14 teams followed Kan et al. (2003) [6] or Jepsen et al. (2018) [34] [16,17,18,20,21,22,23,25,26,28,30,31,32,33]. The gingiva was classified as thin if the graduated periodontal metal probe was visible through the marginal gingiva (implying that its thickness was less than 1 mm), and it was thick otherwise.

Peixoto et al. (2015) [19] described three gingival phenotypes (thin, intermediate, and thick) based on the results of De Rouck et al. (2009) [35]. The gingival phenotype was described as thin when the periodontal probe was visible through the gingival margin in both maxillary central incisors, intermediate when the probe was visible only in one incisor, and thick if the probe was not visible in both incisors. Nik-Azis et al. (2023) [28] also described several types of gingival phenotype following the recommendations of Rasperini et al. (2015) [36]; they were described as very thick if the tips of all three plastic probes (blue, green, white) were not visible during probing, thick if only the blue tip was visible, medium if only the blue and green tips were visible, and thin if all tips were visible.

When gingival thickness was assessed using the endodontic file test, Alhajj (2020) [24] considered the gingiva to be thin if its thickness was less than 1.5 mm, and thick if it was greater than 2 mm. Thickness was not categorized if it was between 1.5 and 2 mm.

The height of the gingival tissue, which is by definition keratinized, was evaluated in millimeters by 11 teams out of 17 using a graduated periodontal probe [18,19,21,22,23,24,25,26,27,31,32], with additional visualization of the mucogingival line using iodine solution for Lee et al. (2018) [23] and Shah et al. (2015) [18].

Alhajj in 2020 [24] was the only one to determine three values: ≤4 mm, between 4 and 8 mm, and >8 mm.

In addition, no author linked the height of the keratinized tissue with a diagnostic criterion to enable the quantity of the gingiva to be described as reduced or high.

In addition, some studies also considered papillae size [17,19,21,22,24,30,31,32,33], and/or tooth morphology [17,19,24,25,27,30,31,32,33], or even gingival pigmentation for the team of Nik-Azis et al. (2023) [28].

- Information about the investigators.

In 16 studies, all mucosal and dental parameters were recorded by one or two examiners. However, only six teams specified the intra-examiner reliability, which was always satisfactory as it ranged from 0.98% to 73% [16,25,27,28,30,33]. Peixoto et al. (2015) [19] did not provide any information on the investigator(s) who collected the clinical data.

### 3.3. Main Results

The main results are summarized in Table 3.

- Overall assessment of gingival thickness, for all subjects, all types of teeth, and all means of assessment combined.

For all individuals, 10 teams found a higher prevalence of a thick gingiva [16,17,18,19,20,27,28,30,31,33]. It should be noted that Shao et al. (2018) [31] and Nik-Azis et al. (2023) [28] were able to confirm this result, whether the thickness of the gingiva was assessed using the probe transparency technique or via transgingival probing. In contrast, four teams reported a higher prevalence of a thin gingiva [22,23,25,32]. Only one team mentioned an equal distribution between the two gingival groups [21]. In addition, we were unable to assess the prevalence of gingival thickness for two studies, as either the results were provided with teeth as reference units [26], or the categorical classification used by the authors was specific [24]. For Alhajj (2020) [24], the majority of subjects in their source population had gingiva thickness ranging from 1.5 to 2 mm (intermediate category, depending on the evaluation criterion chosen).

- Assessment of gingival thickness according to dental arch, all types of teeth, and means of assessment combined (Table 4).

For the maxilla, nine teams indicated, using the probe transparency technique, that the prevalence of a thick gingiva was significantly higher than that of a thin gingiva [16,17,19,20,26,27,28,30,33]. When the transgingival probing technique was used, the results were balanced between the two dental arches [18,23]. Overall, more studies showed a higher prevalence of dental sites with a thick gingiva.

For the mandible, only the study by Fischer et al. (2022) [26] could be considered, and this showed a higher percentage of dental sites with thin gingiva, contrary to what the authors indicated for the maxilla. The other two teams who studied mandibular dental sites only provided global results and not arcade by arcade [24,31].

Finally, we were unable to compare the prevalence of the gingival phenotype between the two dental arches due to a lack of results for the mandibular dental sites.

- Assessment of gingival thickness as a function of tooth type, regardless of the method used (Table 5).

In the maxilla, when gingival thickness was assessed using the probe transparency test, seven studies showed a higher percentage of a thick gingiva in the maxillary central incisors [16,17,19,27,28,30,33], in contrast to three others [26,27,32]. For the other tooth types, Fischer et al. (2022) [26] found that the gingiva was rather thin on the premolars and mostly thick on the first molars.

When the periodontal probe transparency test was performed in parallel with the transgingival probing, Lee et al. (2018) [23] provided conflicting results for central incisors. On the other hand, these authors reported comparable results with both methods for lateral incisors, canines, premolars, and first molars: the gingiva was mostly thick for molars and rather thin for the other teeth.

For the mandible, two studies reported converging results: the gingiva was thin on all incisors and premolars and thick on the first molars [23,26].

Ultimately, the gingiva appears to be thickest on the first molars, whether in the maxilla or mandible. For the central incisors, the gingiva is thicker in the maxilla and thinner in the mandible. For the other types of teeth, the gingiva is more often thin in the maxilla and mandible.

- Overall assessment of the height of the gum (or keratinized tissue).

Only Alhajj (2020) [24] assessed this anatomical parameter in relation to the subjects, who were divided into three groups according to the height of their keratinized tissue. The author found that 70% of the subjects had a gum height of between 4 and 8 mm, 25% less than or equal to 4 mm, and 5% greater than 8 mm.

- Association of gingival thickness and height of keratinized tissue (HTK).

A correlation between the height of keratinized tissue and gingival thickness was shown in three studies, but this result only concerned maxillary central incisors.

Fischer et al. (2015) [21] and Singh et al. (2016) [22] found that this association was significantly positive with a thick gingiva. Collins et al. (2021) [25] determined that this association was statistically positive with a thin gingiva. All of these authors assessed gingival thickness in the same way by using the periodontal probe transparency test with the PCP UNC 15 probe.

Alhajj (2020) [24] divided the subjects into three groups according to the height of the keratinized tissue, and he concluded that there was a significantly positive association between a thin gingiva (thickness < 1.5 mm) and an intermediate height of keratinized tissue (between 4 and 8 mm), whatever the type of tooth.

### 3.4. Secondary Results

- Association between gingival thickness and gender.

Twelve teams examined this association. The results of six of them showed an unequal distribution of gingival phenotypes according to gender; more specifically, the authors described a majority of men with thick gums and a majority of women with thin gums [16,17,21,30,32,33]. However, these results were only significant for the study by Zawawi et al. (2012) (*p* = 0.001) [16]. For the other six studies, no association was found [18,19,23,24,25,26].

- Association between gingival thickness and papillae height.

Only the team of Fischer et al. (2015) [21] looked into this question, and they indicated a significant correlation between these two clinical parameters in the maxillary central incisors. The thinner the gingiva is, the lower the height of the papillae is, and vice versa.

- Gingival thickness/dental morphology association.

Some authors also sought to study the link between dental morphology and gingival phenotypes. The main clinical parameter used was the ratio between the width and coronal length of the teeth studied.

Alhajj (2020) [24] found that rectangular maxillary central incisors were predominantly associated with thin gingiva and square maxillary central incisors with gingiva thicknesses between 1.5 and 2 mm (intermediate category, according to the author). Shetty and Bhat (2013) [17] determined that a thick gingiva was significantly associated with short and wide maxillary central incisors, whereas a thin gingiva was predominantly associated with long and narrow teeth. However, for Fischer et al. (2014) [30], gingival thickness was not related to tooth shape.

- Gingival thickness/smoking association.

The link between tobacco consumption and gingival phenotype was only assessed by two teams. Zawawi et al. (2012) [16] classified subjects into three groups according to their smoking status: active smokers, ex-smokers, and never-smokers. These authors found that the number of active smokers with a thick gingiva was significantly greater than the other two groups (*p* = 0.011). However, Alhajj (2020) [24] did not confirm these results, but he was able to demonstrate a significant correlation between smoking for less than 5 years and the presence of thin gingiva, regardless of daily tobacco consumption (*p* = 0.007). In other words, when the number of years of smoking is low, the gums do not thicken.

- Association with the dental angle class.

In the study by Zawawi et al. (2012) [16], a higher tendency for angle class 1 was shown in men with thick gingiva on their maxillary incisors, but the association was not significant (*p* = 0.08).

- Association with bone morphology.

The association between the thickness of the gingiva and that of the vestibular bone table was only studied by two teams and only in the vestibular region of the maxillary incisors. Frost et al. (2015) [20] found a positive correlation between the thickness of the gingiva and that of the bone table and vice versa, but the results provided were not significant (*p* = 0.06). However, Joshi et al. (2017) [32] statistically confirmed this positive correlation in both the male (*p* < 0.01) and female (*p* < 0.01) groups.

- Association with gingival pigmentation.

In 2023, Nik-Azis et al. [28] found that subjects with pronounced gingival pigmentation, classified as DOPI scale 2 and 3 (Dummet Oral Pigmentation Index), were four times more likely to have a non-visible periodontal probe transparency test (*p* < 0.001). Nevertheless, it seems logical that probe visibility is more difficult to interpret when the gingiva is colored by melanin pigments.

- Association with the smile line.

The results of the Rodrigues et al. (2022) [27] study showed a significant positive association between the thick gingival phenotype, assessed using the periodontal probe transparency test, and the position of the smile line: the more teeth visible when smiling, the thicker the gingiva.

- Geographical origin of populations.

Of the ten studies from Asia, six showed a higher prevalence of a thick gingiva [16,17,18,28,31,33]. However, these studies concerned only 27% of the Asian subjects in all the included studies (738 subjects/2728 in total).

On the European continent, three studies also showed a higher prevalence of a thick gingiva (100 subjects out of 186 for all the studies) [19,26,30], unlike the study by Fischer et al. 2015 [21], which showed an equal distribution between the two types of gingiva.

As regards the three studies from the American continent, one concluded that the majority of subjects had thin gums [25], and the other two were the opposite, with a greater number of subjects having thick gums [20,27].

So, all things considered, given the uneven number of studies among the different continents, it is not possible to determine the existence of a correlation between gum thickness and geographical location.

### 3.5. Assessing Bias in Studies

The type of studies included was similar; they were cross-sectional descriptive studies, designed to assess the prevalence of an event.

For the 17 studies included, the MMAT score ranged from 0%–40% (very high or high risk of bias) to 60% (moderate risk of bias), with a mean risk of bias of approximately 37% and a standard deviation of 13%. Inter-examiner agreement for the assessment of the main risks of bias was judged to be high (k = 0.81).

More specifically, all the studies had a very high or high risk of bias, except for Zawawi et al. 2012 [16], which had a score of 60% (Table 6).

Several types of bias can be cited:

1. Selection bias (questions 1 and 4 of the MMAT tool): non-probabilistic or unspecified recruitment method; samples poorly distributed in relation to individual criteria; no information on the number or individual characteristics of patients who refused inclusion.
2. Confounding bias (question 2 of the MMAT tool): periodontal condition not precisely indicated; known risk factors that can bias the assessment of gingival thickness, such as smoking, a physiological hormonal factor, or medication that causes gingival growth, are not taken into account.
3. Classification bias (questions 3 and 5 of the MMAT tool): unspecified or unsuitable measurement instrument; uncalibrated examiner; no blinding; unspecified statistical method.

## 4. Discussion

- Assessment of the prevalence of gingival phenotypes.

Clinically, periodontal health can be defined as a functional periodontal state, free of any inflammatory disease or lesion, and associated with a feeling of comfort or well-being [37,38]. This holistic, patient-centered approach is not only of semantic or didactic interest; it allows clinicians, teachers, and researchers to establish anatomophysiological criteria specific to health or a pathological state, enabling the greatest number of people to optimize diagnostic and therapeutic approaches, particularly periodontal. Among these criteria, the gingival phenotype is considered an essential clinical determinant, as it reflects the extent of physiological anatomical variations in the gingiva, both within and between individuals. Its assessment enables us to anticipate the behavior of gingival tissue in the face of aggression of any kind.

We therefore felt it was important to know the prevalence of different gingival phenotypes, particularly in healthy adults with a healthy or restored superficial periodontium. This is why we conducted this systematic review, the main objective of which was to assess the current value of clinical data for this type of subject. Indeed, the main reviews available in recent years have focused on the effects of risk indicators/factors associated with gingival phenotype [13,39], and their main objective was not to determine its prevalence.

Thus, after critical analysis of the 17 eligible reviews, we concluded that the prevalence of the gingival phenotype cannot be correctly assessed without taking into account the reference unit. In fact, no team examined the gingival phenotype for a complete dentition. This anatomical characteristic was only partially assessed for each individual in the source populations studied, i.e., only for certain dental sectors, which were not chosen at random. The most studied dental sectors were the incisors and maxillary first molars. The least studied were the mandibular premolars and incisors. A comparison of the results of the selected studies also indicates that gingival thickness differs between maxillary and mandibular sites. Consequently, to date, there is no epidemiological study that can accurately determine the prevalence of the gingival phenotype on an individual basis, i.e., for a complete dentition. Even the classification of Zweers et al. (2014) [2], which distinguishes three main periodontal morphotypes (flat/thick, scalloped/fine, and scalloped/thick) does not allow this, as it was developed from studies presenting assessments carried out only at the level of certain teeth.

**Figure 2:**
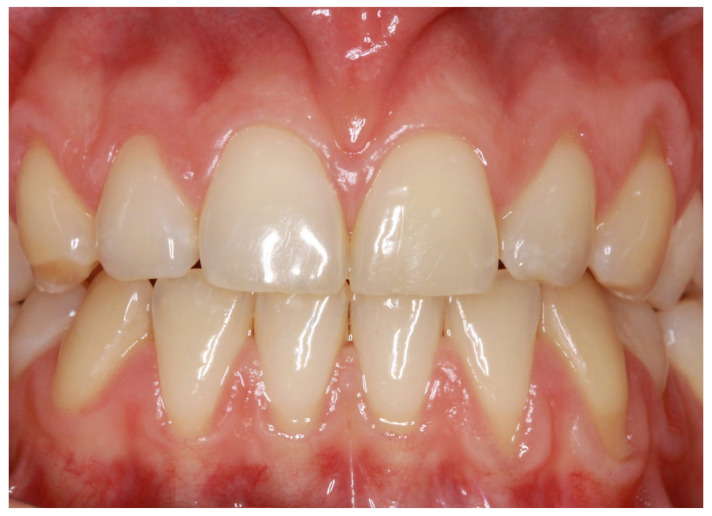
Clinical example illustrating the variability of the gingival phenotype depending on the dental sector concerned (thick in the maxillary anterior sector and thin in the mandibular anterior sector).

In everyday practice, this notion is important to consider. An initial estimate of an individual’s gingival morphology, for example, at the time of the first consultation, is entirely possible via the classification of Zweers et al. (2014) [2], which is nonetheless useful. However, when periodontal/implant, orthodontic, or prosthetic surgical treatment is required, it no longer seems appropriate. In such cases, only a precise assessment of the gingival phenotype, using the probe transparency test or transfixing probing, at dental sites judged visually to be at risk, seems relevant, as it is adapted to the therapeutic objectives. This second approach allows us to better appreciate the physiological diversity of the gingival phenotype, which is not necessarily homogeneous from one maxilla to another, from one dental sector to another, or from one tooth to another [39] (Figure 2). In this respect, the other clinical notion highlighted by this review is that the thick gingival phenotype is mainly found in maxillary incisors and maxillary and mandibular first molars. For other tooth types, the results are less conclusive because the number of clinical studies devoted to them is limited. However, preliminary results suggest that the gingiva is rather thin in the mandibular incisors and premolars [23,26], which would partly explain why these dental sectors are considered to be high-risk sites for gingival recession [40].

Furthermore, gingival height cannot be considered as a predictor of gingival thickness. On the one hand, this is because studies assessing the link between these two clinical parameters only concern maxillary anterior teeth, and on the other, their results show that high gingiva can be either thick or thin.

In clinical practice, it therefore seems logical to assess these two anatomical features independently of each other.

All these results are in line with the conclusions of the two systematic reviews that preceded ours and whose objective was precisely to evaluate this type of link [12,39].

- Indicators/risk factors for gingival phenotypes.

Regarding the risk factors that may influence the gingival phenotype, the low level of evidence in the studies included and their heterogeneity (in terms of the population or dental site studied and the methods used to discriminate the gingival phenotype) prevent us from giving reliable clinical guidelines. In fact, the conclusions of the various authors can only generate hypotheses, as they are not supported by scientific evidence.

Certain trends can nevertheless be identified.

Cigarette smoking and a gummy smile appear to be associated with a thicker gingival phenotype. With regard to smoking, this relationship could be explained by the phenomenon of reactive fibrosis that affects the gingival chorion of smokers. However, it is not possible to link this trend to a dose or time effect of smoking, as the number of studies devoted to this issue is too small [16,24].

In contrast, gender, dental morphology or vestibular bone table morphology, and skeletal angle class do not appear to be reliable risk indicators for predicting the type of gingival phenotype. For gender and dental morphology, our results concur with the conclusions reached by Kim et al. (2020) [39]. The other clinical data do not agree with those recently determined by Shafizadeh et al. (2022) [13] and Kong et al. (2023) [41]. This is perhaps due to the fact that the included studies dealing with these criteria were only selected in relation to our main objective. The meta-analysis by Shafizadeh et al. (2022) [13] shows a significant association between a thick gingival phenotype and a thick vestibular bone table, particularly in the crestal region. For their part, in their cross-sectional study of 177 subjects, Kong et al. (2023) [41] documented a significant association between a thin phenotype and the normodivergent and hypodivergent groups. However, it is important to note that these authors assessed the gingival phenotype only in the mandibular central incisors.

Finally, as regards the geographical origin of the subjects, we were unable to establish a link between this and the thickness of the gingiva. There are two possible reasons for this. The first concerns the uneven number of studies among the different continents. It is interesting to note that no trials were conducted in Africa or Oceania. The second concerns the sampling protocols for individuals, which did not take into account the ethnic groups of the source populations (endogenous or immigrant groups?).

However, an individual’s ethnic origin may be more important than their country of origin when assessing the gingival phenotype, as this individual variable may condition the characteristics of the gingival tissue (melanin coloration, growth of bone bases, for example) or lifestyle habits (diet, for example). Our results do not contradict those of Hsu et al. (2020) [42], who showed in their cross-sectional study that the Asian American subjects they studied were more likely to have thin gums than the Black American subjects. In this study, the subjects were all of immigrant origin, but only their continental origin was mentioned. In fact, the homogeneity of the two groups was not determined by the authors, who did not specify the different ethnic groups. In other words, the external validity of this study is limited.

- Limitations.

Finally, this systematic review has a major limitation in that it was not possible to carry out a statistical analysis of the results, due to the differences in the quality of the internal validity of the studies (sampling methods for individuals and dental sites, means of assessment, and criteria for judging the gingival phenotype).

However, it does highlight the lack of scientific resources concerning the prevalence of gingival phenotypes and associated risk factors.

- Clinical relevance.

When a clinician, whether a general practitioner or specialist, must take into account the characteristics of the gum tissue before undertaking surgical, orthodontic, or prosthetic treatment, they must always remain vigilant without relying on the overall appearance of the dentition and independently assess the thickness and height of the gum at the dental sites where they wish to intervene. A simple analogical visual inspection overlooks physiological tissue variations. On the other hand, a more reasoned, analytical diagnostic approach allows clinicians to be more attentive and encourages them to supplement their visual inspection with a targeted clinical examination. This will enable them to establish precisely the type of gingival phenotype at sites initially considered to be at risk. This wise professional attitude strengthens clinicians’ skills and limits the risk of anchoring bias [43], which defines the tendency to remain focused on one’s first clinical impression, to the detriment of other more relevant and useful information to avoid diagnostic and therapeutic errors.

In practice, therefore, it is possible to accurately assess the gingival phenotype at a single site by relying on the threshold values classically accepted by most periodontic experts: the gingiva is said to be thick (>1 mm) if the CPN15 periodontal probe cannot be seen through the marginal gingiva; otherwise, it is said to be thin. The gingiva is said to be high if its height is ≥2 mm and reduced otherwise [1]. All combinations are possible, depending on the individual data specific to each person and tooth type, bearing in mind that a reduced and thin gingiva represents a mucosal environment that is not very resistant to aggression of all kinds.

## 5. Conclusions

There is insufficient data in the literature to establish the prevalence of the gingival phenotype on an individual scale. Only a few studies suggest that there are a small number of individuals with a homogeneous overall phenotype, i.e., consistently thick/fine or high/reduced throughout the dentition. On the other hand, this systematic review provides the clinician with information on the prevalence of this gingival phenotype at different dental sites. The maxillary central incisors and maxillary or mandibular first molar sectors are readily associated with a high and thick gingival phenotype, independently of the dental morphology, gender, and age of adult subjects. Furthermore, in these regions, this gingival phenotype tends to be associated with a thick vestibular bone table. In contrast, maxillary and mandibular incisors and premolars more often have a thin gingival phenotype. These dental sites are more vulnerable to loss of attachment. It is therefore advisable to analyze their gingival phenotype in detail before considering orthodontic, prosthetic or implant treatment, or long-term periodontal follow-up. In such circumstances, periodontal plastic surgery may be indicated to thicken and/or increase the height of the attached gingiva.

Further longitudinal studies are needed in the future. For reasons of feasibility and pragmatism, we believe that they should be designed according to therapeutic needs, dental sector by dental sector, and within homogeneous source populations.

## Figures and Tables

**Table 1:** Search equations based on electronic databases.

Database	Search Strategy
PubMed (MEDLINE)	((“healthy adults”) OR (“periodontal health”)) OR (“general population”)) OR (“epidemiological”)) OR (“clinical study”)) OR (“human study”))) AND ((“gingival biotype”)) OR (“periodontal biotype”)) OR (“gingival phenotype”)) OR (“periodontal phenotype”)) OR (“gingival width”)) OR (“gingival thickness”)) OR (“thick biotype”)) OR (“thin biotype”)))
The Cochrane Library	(“Periodontium” OR “Gingiva”) AND (“Morphotype” OR “Thickness” OR “thick” OR “Thin” OR “Biotype” OR “Flat”)
Embase	(“healthy adults” OR “periodontal health” OR “general population” OR “epidemiological” OR “clinical study” OR “human study”) AND “gingival biotype” OR “periodontal biotype” OR “gingival phenotype” OR “periodontal phenotype” OR “gingival width” OR “gingival thickness” OR “thick biotype” OR “thin biotype”)
Scopus	(KEY (periodontal AND phenotype) OR KEY (gingival AND biotype) OR KEY (gingival AND thickness) AND KEY (prevalence))

**Table 2:** General characteristics of included studies. M = male, F = female, CBCT = Cone Beam Computer Tomography, PD = pocket depth, AG = attached gingiva, max = maxillary, mand = mandibular, MG = marginal gingiva.

Authors Year of Publication	Objectives	Population/Types of Teeth/Recruitment	Inclusion/Exclusion Criteria	Materials and Methods
Zawawi et al. 2012 [16]	Primary: To assess the prevalence of gingival biotypes, and the association with the different types of malocclusions. Secondary: Assess the relationship with the smoking habits (current smoker/former smoker/never smoker).	200 subjects (100 M, 100 F) 50% F (average age:31.7 years)/50% M (average age: 32.4 years) Max central incisors Recruitment: random successive mode at the dental faculty of King Abdulaziz University in Jeddah, (Kingdom of Saudi Arabia)	Inclusion criteria: 18 years or older Presence of all max anterior teeth Exclusion criteria: Dental crowns History of an orthodontic treatment Antibiotic treatment required for the examination Medicinal treatment with a known effect on the periodontal soft tissues, pregnant or breastfeeding women	Parameters recorded: Gingival thickness Dental occlusion (angle classification or canine relationship) Tobacco consumption Protocol: A single calibrated examiner (superior reliability 93%) Williams periodontal probe (Hu-Friedy^®^, Chicago, IL, USA) Transparent probe technique
Shetty and Bhat 2013 [17]	Primary: To assess gingival thickness (biotype). Secondary: Evaluate the prevalence of gingival biotypes of max central incisors as a function of gender, age, tooth shape, and papillary height.	200 subjects (125 M, 75 F) 37.5% F/62.5% M 18–30 age group = 125 subjects, 30–50 age group = 75 subjects Max central incisors Inclusion following a visit to the outpatient department in Mangalore, Karnataka (India)	Inclusion criteria: Age between 18 and 50 Exclusion criteria: Catering/crowns Orthodontic treatment, dental malposition Periodontitis (PD > 3 mm) or recession Pregnant/lactating women	Parameters recorded: Gingival thickness, papillary height Crown length/width ratio Protocol: A single calibrated examiner (kappa not quoted) Type of periodontal probe not quoted Transparent probe technique
Fischer et al. 2014 [30]	Primary: Determine the correlation between the gingival biotype, the supracrestal vestibular, and the interproximal gingival height. Secondary: Assess the correlation between the gingival biotype and the crown shape or the correlation between the gingival biotype and the papilla height.	80 subjects (34 M, 46 F) 57.5% F/42.5% M (mean age 25.8 years) Max central incisors Inclusion in the Julius-Maximilians University in Wuerzburg (Germany) without further details	Inclusion criteria: Presence from 13 to 23 Exclusion criteria: Restoration/crown, severe attrition Periodontitis (PD > 3 mm) or recessions Medicinal treatment with a known effect on periodontal soft tissues, pregnant/lactating women, bone disease Smoking (>10 cigarettes/day)	Parameters recorded: Gingival thickness, papillary height Gum height Crown length/width ratio Protocol: A single calibrated examiner (97% reliability) UNC-15 periodontal probe (Hu-Friedy^®^, Chicago, Il, USA)Transparent probe technique
Shah et al. 2015 [18]	Primary: To assess the prevalence of gingival biotypes. Secondary: Evaluate the relationship between the gender, the recessions, and the gum height.	400 subjects (200 M, 200 F) 50% F/50% M (mean age 28.82 years) Max central and lateral incisors, canines Recruitment: not specified (India)	Inclusion criteria: Good general health, no dental crowding Exclusion criteria: Removable prosthesis, orthodontic device Absence of one of the six max anterior teeth Periodontal recessions (class III/IV Miller) History of smoking or mouth breathing	Parameters recorded: Gingival thickness, gingival height (with iodine solution) PD, gingival recession Protocol: Single blind calibrated examiner (kappa not quoted) UNC-15 periodontal probe (Hu-Friedy^®^, Chicago, Il, USA) Transgingival probing (endodontic file)
Peixoto et al. 2015 [19]	Primary: Determine the relationship between the gingival thickness, the papillary height, and the height of the gum Secondary: Assess the relationship with the gender, the shape, and the location of the crown	50 subjects (20 M, 30 F) 60% F/40% M (average age not given) Max central incisors Place and method of recruitment not cited (Portugal)	Inclusion criteria: Good general health, max anterior teeth present Exclusion criteria: Dental malposition, restorations/crowns History of orthodontic treatment, periodontal surgery Periodontitis Medicinal treatment known to have an effect on periodontal soft tissues, pregnant/breastfeeding women	Parameters recorded: Gingival thickness, gingival height Papillary height Crown length/width ratio Gingival angle, gingival asymmetry Protocol: No information about the examiner OMS periodontal probe (Henry Schein^®^, Melville, NY, USA) Probe transparency technique + analysis based on the intraoral photographs
Frost et al. 2015 [20]	Primary: To determine the objective measurement of the gingival thickness in relation to a gold standard (transparency of the probe). Secondary: To correlate with the thickness of the alveolar bone.	56 subjects (33 F, 23 M) 58.9% F/41% M (mean age 53) Max incisors, canines, premolars Inclusion in UTHSCSA Dental School (Texas, USA) unspecified	Inclusion criteria: Age > 18 years No gum disease Presence of at least one maxillary anterior tooth (incisors), one canine, one first PM Exclusion criteria: Restoration, dental malposition, gingival hypertrophy History of orthodontic treatment or surgery Periodontitis (PD ≥ 4 mm) Uncontrolled systemic disease Drug treatment with a known effect on periodontal soft tissues, pregnant women	Parameters recorded: Gingival biotype, gingival thickness Thickness of the bone table Protocol: UNC-15 periodontal probe (Hu-Friedy^®^, Chicago, IL, USA) Probe transparency technique (gold standard) + intra-oral photographs (three examiners) Transgingival probing (endodontic file) + intraoral photographs (one examiner) CBCT
Fischer et al. 2015 [21]	Primary: Assess the association between the gingival biotype and the gingival thickness. Secondary: Compare and analyze the extreme biotypes (very thin, very thick).	36 subjects (19 F, 17 M) 53% F/47% M (average age 24.9) Max central incisors Inclusion in the Julius-Maximilians University in Wuerzburg (Germany) without further details	Inclusion criteria: Presence of teeth, 13 to 23 Exclusion criteria: Restoration/crown, crowding/dental malposition Periodontitis (PD > 3 mm) or recessions Drug treatment with a known effect on the periodontal soft tissues, pregnant/lactating women Smoking (>10 cigarettes/day)	Parameters recorded: Gingival thickness, gingival height Papillary height PD Protocol: A single calibrated examiner (kappa not quoted) UNC-15 periodontal probe (Hu-Friedy^®^, Chicago, IL, USA) + digital caliper Probe transparency technique
Singh et al. 2016 [22]	Primary: Assess the correlation between the gingival thickness, the gingival height, PP, and the papillary height.	363 subjects, sex distribution not given (mean age not given) Max central, lateral, and canine incisors Inclusion in the Navi Mumbai Dental College (India) without further details	Inclusion criteria: Over 18 years of age Presence of maxillary anterior teeth Exclusion criteria: Low lip-brake attachment, restorations/crowns Orthodontic treatment or history of periodontal surgery Medicinal treatment with a known effect on periodontal soft tissues, self-mutilation	Parameters recorded: Gingival thickness, gingival height PD, papillary filling Protocol: One single calibrated examiner (kappa not quoted) UNC-15 periodontal probe (Hu-Friedy^®^, Chicago, IL, USA) Transparent probe technique
Joshi et al. 2017 [32]	Primary: Assess and compare the gingival biotype by gender. Secondary: Evaluate the relationship between the gingival thickness and the alveolar bone thickness according to gender.	800 subjects (400 M, 400 F) 50% F, 50% M (average age 22/21 years) Max central incisors Inclusion in the Department of Periodontology, School of Dental Sciences (India), unspecified	Inclusion criteria: Presence of teeth, from 13 to 23 Exclusion criteria: Restoration/crown, cervical attrition Periodontitis (PD ≥ 4 mm) or recessions Drug treatment with known effect on periodontal soft tissues, pregnant/lactating women Systemic disease (gingival or bone manifestation)	Parameters recorded: Gingival thickness (clinical and radiographic), gingival height Papillary height (photographic measurement) Crown width/length ratio (photographic measurement) Alveolar bone thickness (radiographic) Protocol: A single calibrated examiner (kappa not quoted) UNC-15 periodontal probe (Hu-Friedy^®^, Chicago, IL, USA) Transparent probe technique
Lee et al. 2018 [23]	Primary: Determine the gingival biotype of the teeth and the association with age, ethnicity, gender, the type of teeth, the presence of plaque, and the recessions. Secondary: Evaluate the concordance of the gingival thickness assessment methods (probe transparency/transgingival probing).	51 subjects (24 M, 27 F) 53% F/47 M (average age 30.3 years) Max and mandibular incisors, canines, first and second premolars, first molars Inclusion in the Singapore National Dental Centre without further details	Inclusion criteria: 21 years or older Healthy or reduced periodontium Bleeding scores ≤ 15% Exclusion criteria: Orthodontic treatment, restoration less than 1 mm from the marginal gingiva, dental crowding, dystopia/ectopia Previous periodontal surgery Uncontrolled systemic disease, allergy to iodine solution Drug treatment with a known effect on periodontal soft tissues, pregnant/lactating women	Parameters recorded: Gingival thickness, gingival height Gingival recession Bleeding on probing Protocol: A single calibrated examiner (kappa not quoted) UNC-15 periodontal probe (Hu-Friedy^®^, Chicago, IL, USA) Transparent probe technique Transgingival probing (endodontic file) Use of an iodine solution
Shao et al. 2018 [31]	Primary: Assess the distribution of the periodontal biotype. Secondary: Evaluate the different techniques for assessing gingival thickness (probe transparency/transgingival/CBCT).	31 subjects (15 M, 16 F) 51% F/49% M (mean age 22.2 years) Max and mandibular incisors and canines Inclusion in the College of Stomatology at Nanjing University (China), unspecified	Inclusion criteria: Age between 18 and 30 years No gingival index ≤ 1, PD ≤ 3 mm, no loss of attachment ≥ 1 mm No radiological signs of alveolar lysis, no malocclusion, no crowding, no supernumerary teeth Presence of anterior teeth Exclusion criteria: Restoration/crown, orthodontic appliance Previous periodontal surgery Uncontrolled systemic disease Medicinal treatment with a known effect on periodontal soft tissues, pregnant or breastfeeding women Smoking, bruxism	Parameters recorded: Gingival thickness, gingival height, and AG Height of papillae Crown length/width ratio PD Protocol: A single calibrated examiner (kappa not quoted) Williams periodontal probe (Hu-Friedy^®^, Chicago, IL, USA) Probe transparency technique Transgingival probing (endodontic file) CBCT
Alhajj 2020 [24]	Primary: Assess the prevalence of gingival phenotypes. Secondary: Assess the correlation between age, gender, and tobacco and khat consumption.	456 subjects (215 M, 241 F) 53% F/47% M (mean age 29.9 years) Max and mandibular central and lateral incisors and first molars Inclusion in the private clinic in the city of Sanaa (Yemen) without further details	Inclusion criteria: Good general health No dental crowding Exclusion criteria: Oral ventilation, restoration/crown Removable appliance (partial prosthesis or orthodontic) Absence of one of the six max anterior teeth Recessions (Miller class III/IV) Drug treatment with a known effect on periodontal soft tissues, pregnant/lactating women	Parameters recorded: Gingival thickness, gingival height Height of papillae Crown width/length ratio Protocol: A single calibrated examiner (kappa not quoted) UNC-12 periodontal probe (Hu-Friedy^®^, Chicago, IL, USA) Transgingival probing (endodontic file)
Yin et al. 2020 [33]	Primary: Correlate the periodontal biotype and the clinical parameters of the gingiva and the crown.	56 subjects (13 M, 43 F) 77% F/23% M (average age 23.6) Max right central incisor Inclusion in the campus of the School of Stomatology at Shandong University (China) without further details	Inclusion criteria: Age between 18 and 40 years Plaque index < 1, gingival index < 1 No dental malposition or anomaly of shape Presence of all max anterior teeth Exclusion criteria: Dental restoration/cavity Drug treatment with a known effect on periodontal soft tissues Periodontitis (PD ≥ 4 mm) or recession Gingival pigmentation	Parameters recorded: - Gingival thickness, gingival height Gingival angle, width, and height of the papilla Crown length and width Bucco-lingual width of the crown Width and height of contact surface Protocol: Two calibrated examiners (kappa: 0.733) William periodontal probe (Hu-Friedy^®^, Chicago, IL, USA) Transparent probe technique + intraoral photographs
Collins et al. 2021 [25]	Primary: Assess the prevalence of gingival phenotypes. Secondary: Assess the association with the other clinical and demographic variables.	107 subjects (63 M, 44 F) 41% F/59% M (mean age 30.7 years) Max central incisors Recruitment: voluntary work in eight districts of Santo Domingo (Dominican Republic) with no further details available	Inclusion criteria: 18 years and over Good general health Presence of at least the max central incisors Exclusion criteria: Restoration/veneer, crowding > 3 mm Periodontitis and/or gum recession History of periodontal surgery Fixed or removable orthodontic appliance Attrition of more than a third of the crown Medicinal treatment with a known effect on periodontal tissues, pregnant or breast-feeding women	Parameters recorded: Gingival thickness, gingival height, and AG PD Dental morphology (Gobbato classification) Protocol: A single calibrated examiner (98% agreement) UNC-15 periodontal probe (Hu-Friedy^®^, Chicago, IL, USA) Transparent probe technique
Fischer et al. 2022 [26]	Primary: Screening for gingival biotype on different teeth. Secondary: Evaluate the association with gender.	56 subjects (20 M, 36 F) 64% F/36% M (average age 23) Teeth: 16, 21, 24, 36, 41, 44 Inclusion in the University of Witten/Herdecke (Germany) without further details	Inclusion criteria: Not indicated Exclusion criteria: Restoration/crown Crowding or malposition Periodontitis (PD ≥ 3 mm) or recessions Drug treatment with a known effect on periodontal soft tissues, pregnant women Smoking (>10 cigarettes/day)	Parameters recorded: Gingival thickness + gingival height PD Protocol: A single calibrated examiner (kappa not quoted) PCP12 periodontal probe (Deppeler SA^®^, Rolle, Switzerland) Transparent probe technique
Rodrigues et al. 2022 [27]	Primary: Assess the correlation between the smile type and the periodontal phenotype.	164 subjects (48 M, 116 F) 30% M/70% F (average age 23) Maxillary central incisors (328 teeth) Inclusion in the dental school of the Federal University of Fluminense (Rio de Janeiro, Brazil), unspecified	Inclusion criteria: 18 years and over Presence of intact maxillary anterior teeth (central and lateral incisors, canines) Exclusion criteria: History of periodontal surgery in the anterior maxilla Periodontitis or recessions History of orthodontic treatment Drug treatment with a known effect on periodontal soft tissues, pregnant or breastfeeding women Systemic disease Tobacco consumption Gingival smile (greater than 3–4 mm) Symptoms of facial paralysis Cosmetic procedure on the upper lip	Parameters recorded: Gingival thickness, gingival height Gingival architecture Crown length and width Width and height of contact surface Protocol: Photographs: two calibrated examiners (kappa: 0.955). Gingival phenotype: a single examiner (kappa: 0.967). Tomography: a single examiner (kappa > 0.895) UNC-15 periodontal probe (Hu-Friedy^®^, Chicago, IL, USA) Transparent probe technique + intraoral photographs + tomographic measurements
Nik-Azis et al. 2023 [28]	Primary: Compare the probe visibility method with the direct caliper measurement for measuring gingival thickness. Secondary: Compare the gingival measurements in subjects with different levels of gingival pigmentation.	171 subjects (45 M, 126 F) 26% M/74% F (average age 25) Max right central incisor (171 teeth)Recruitment of students, staff, and patients from the UKM Faculty of Dentistry (Malaysia) without further details	Inclusion criteria: 18 years and over Good general health Presence of upper central incisors Healthy periodontium Exclusion criteria: Restoration/crown on max central incisors Periodontitis or recessions Medicinal treatment with a known effect on periodontal soft tissues, pregnant or breastfeeding women Smoking	Parameters recorded: Gingival thickness Gingival pigmentation Protocol: Two calibrated examiners (kappa = 0.694 and 0.667) UNC-15 periodontal probe and colored plastic probe (Hu-Friedy^®^, Chicago, IL, USA) Transparent probe technique + transgingival technique (endodontic file) + direct measurement with a caliper

**Table 3:** Main results of included studies. M = male, F = female, n = number of subjects, CBCT = Cone Beam Computer Tomography, HTK = height of keratinized tissue.

Authors Year of Publication	Prevalence	Other Results
Zawawi et al. 2012 [16]	Thick biotype (F and H groups): 111 teeth (55.5%) (probe not visible) Thin biotype (F and H groups): 89 teeth (44.5%) (visible probe) Thick biotype (group H): n = 75 (75%) Thick biotype (group F): n = 36 (36%)	Gums significantly thinner in women. No difference between gingival biotype and malocclusions. No difference between gingival biotype and smoking status.
Shetty and Bhat 2013 [17]	Thick biotype (F and H groups): 108 teeth (54.75%) probe not visible Thin biotype (F and H groups): 92 teeth (45.25%) visible probe Thick biotype (group H): n = 79 (63%) Thick biotype (group F): n = 30 (41%)	Participants with short, wide teeth: 56% have a thick gingival biotype. Participants with long, narrow teeth: 39% have a thick gingival biotype.
Fischer et al. 2014 [30]	Thick biotype (F and H groups): 42 teeth (53%) probe not visible Thin biotype (H and F groups): 38 teeth (47%) visible probe Thick biotype (group H): n = 20 (47%) Thick biotype (group F): n = 22 (52%)	24 F out of 38 have a fine gingival biotype. No correlation between gingival biotype and crown width/length ratio (*p* > 0.05).
Shah et al. 2015 [18]	Thick biotype (H and F groups): 227 teeth (56.75%) greater than 1 mm Thin biotype (H and F groups): 173 teeth (43.25%) less than or equal to 1 mm	Average gingival thickness greater than 1 mm. No correlation between gingival biotype and gender (*p* > 0.05).
Peixoto et al. 2015 [19]	Thick biotype (H and F groups): 28 teeth (56%) probe not visible Intermediate biotype (H and F groups): seven teeth (14%) probe visible on a CI Thin biotype (H and F groups): 15 teeth (30%) probe visible on both ICs Thick biotype (group H): n = 14 (70%) Thick biotype (group F): n = 14 (47%) Intermediate biotype (group H): n = 3 (15%) Intermediate biotype (group F): n = 4 (13%) Thin biotype (group H): n = 3 (15%) Thin biotype (group F): n = 12 (40%)	No correlation between gingival biotype and gender.
Frost et al. 2015 [20]	Thick biotype (H and F groups): 254 teeth (83%) probe not visible Thin biotype (H and F groups): 52 teeth (17%) visible probe Thin premolar biotype: seven teeth/62 (11%) Thin canine biotype: 20 teeth/83 (24%) Thin lateral incisor biotype: 20 teeth/86 (23%) Thin central incisor biotype: five teeth/75 (7%)	Mean gingival thickness significantly smaller for the thin biotype (*p* < 0.001).
Fischer et al. 2015 [21]	Thick biotype (H and F groups): 18 teeth probe not visible Thin biotype (H and F groups): 18 visible probe teeth Thin biotype (group H): n = 7 (39%) Thin Biotype (group F): n = 11 (61%) Thick biotype (group H): n = 10 (56%) Thick biotype (group F): n = 8 (44%)	Significant difference between groups in terms of gingival thickness (*p* < 0.0001), keratinized tissue height (*p* = 0.0371), and papillary height (*p* = 0.0247).
Singh et al. 2016 [22]	Thick biotype (H and F groups): 819 teeth (37.6%) probe not visible Thin biotype (H and F groups): 1359 teeth (62.4%) visible probe	Positive correlation between gingival thickness and height of keratinized tissue.
Joshi et al. 2017 [32]	Thick biotype (H and F groups): 367 teeth probe not visible Thin biotype (H and F groups): 433 teeth visible probe Thin biotype (group H): n = 97 (24.2%) Thin biotype (group F): n = 336 (84%) Thick biotype (group H): n = 303 (75.8%) Thick biotype (group F): n = 64 (16%)	Significant positive correlation between gingival thickness and bone thickness in men and women (*p* < 0.01).
Lee et al. 2018 [23]	Thick maxillary biotype: 134 teeth (36.2%) greater than or equal to 1.5 mm Thin maxillary biotype: 236 teeth (63.8%) smaller than 1.5 mm Thin mandibular biotype: 355 teeth (92.4%) Thick mandibular biotype: 29 teeth (7.6%)	Out of 51 patients: 90% of probes visible on maxillary central incisors, 85% on maxillary lateral incisors, 84% on maxillary first premolars, 75% on mandibular central incisors, 85% on mandibular lateral incisors. No significant differences for gender, age, ethnicity, or type of periodontium. Significant difference in gingival thickness between posterior and anterior teeth. Significant difference in HTK between maxillary anterior and posterior teeth. HTK significantly higher for mandibular incisors.
Shao et al. 2018 [31]	Thick biotype: 222/372 teeth (59.68%) probe transparency Thick biotype: 266/372 teeth (71.51%) endodontic file Thick biotype: 303/372 teeth (81.45%) CBCT Thin biotype: 150/372 teeth (40.32%) probe transparency Thin biotype: 106/372 teeth (28.49%) endodontic file Thin biotype: 69/372 teeth (18.55%) CBCT	Kappa value for transgingival technique: 0.24. No consistency between probe transparency technique and CBCT.
Alhaij 2020 [24]	Thick biotype: 69 teeth (15.1%) thicker than 2 mm Thin biotype: 83 teeth (18.2%) less than 1.5 mm thick Uncategorized biotype: 304 teeth (66.7%) between 1.5 and 2 mm thick	HTK lower in men than in women (*p* = 0.006). Correlation between thin gums and HTK between 4.1 and 8 mm. The rectangular shape of the teeth is preferably associated with thin gums.
Yin et al. 2020 [33]	Thick biotype (H and F groups): 39 teeth (69.6%) probe not visible Thin biotype (H and F groups): 17 teeth (30.4%) visible probe Thick biotype (group H): n = 12 (92.3%) Thick biotype (group F): n = 27 (62.7%)	Significant differences in gingival biotypes between H and F (*p* < 0.2). Significant differences in periodontal biotypes between H and F (*p* = 0.043).
Collins et al. 2021 [25]	Thick biotype (H and F groups): 43 teeth (40.2%) probe not visible Thin biotype (H and F groups): 64 teeth (59.8%) visible probe Thick biotype (group H): n = 24 (37.5%) Thick biotype (group F): n = 19 (44.2%)	No significant difference in the gingival biotype according to gender and age. Significantly finer biotype in individuals with square teeth. HTK significantly greater in patients with thin gums (*p* = 0.011).
Fischer et al. 2022 [26]	Thick maxillary biotype: 59% of teeth probe not visible Thin maxillary biotype: 41% of teeth probe visible Thick mandibular biotype: 49.4% of teeth probe not visible Thin mandibular biotype: 50.6% of teeth probe visible Thick molar biotype: 94.6% of teeth probe not visible Thin molar biotype: 5.4% of teeth probe visible Thick incisor biotype: 29.5% of teeth probe not visible Thin incisor biotype: 70.5% of teeth probe visible	Statistically significant distribution between gingival phenotypes in the maxilla and mandible (*p* = 0.001). Thicker gingivae for molars than for other teeth (*p* = 0.006). No correlation between gingival biotype and gender (*p* = 0.722).
Rodrigues et al. 2022 [27]	Thick biotype (H and F groups): 170 teeth (51.8%) probe not visible Thin biotype (H and F groups): 158 teeth (48.2%) visible probe	Significant association between the gingival smile (high, medium, low) and gingival phenotype assessed by transparency of the periodontal probe (*p* = 0.021).
Nik-Azis et al. 2023 [28]	Thick biotype (H and F groups): 138 teeth (80.7%) probe not visible Thin biotype (H and F groups): 33 teeth (19.3%) visible probe Thick biotype (H and F groups): 143 teeth (83.6%) endo file (>1 mm) Thin biotype (H and F groups): 28 teeth (16.4%) endo file (≤1 mm) Thin biotype (H and F groups): 17 teeth (51.1%) with visible white tips Medium biotype (H and F groups): 13 teeth (39.4%) with visible green tip Thick biotype (H and F groups): 3 teeth (9.1%) visible blue tip Very thick biotype (H and F groups): 0 teeth (0%) no visible tips	Significant correlation between gingival measurements with the caliper and the transgingival method (*p* = 0.003). Subjects with a high level of gingival pigmentation were more likely to have thickened gums.

**Table 4:** Prevalence of thick gingiva per arch, all tooth types combined, according to probe transparency test or transgingival probing. n = number of subjects, F = female, M = male, ND = not determined. The percentage according to gender is determined in relation to the overall percentage. Prevalences > 50% are shown in bold type.

Authors Country	Number of Subjects (F, M), Number of Teeth	Number and Percentage of Teeth with a Thick Gingiva (F, M)
**Maxillary arch**		
Periodontal probe transparency test		
Singh et al., 2016 India [22]	n = 363, 2178 teeth	819 teeth, 37.6% (ND)
Joshi et al., 2017 India [32]	n = 800 (400 F, 400 M), 800 teeth	367 teeth, 45.9% (17.5%, **82.5%**)
Collins et al., 2021 Dominican Republic [25]	n = 107 (44 F, 83 M), 107 teeth	43 teeth, 40.2% (44%, **56%**)
Fischer et al., 2015 Germany [21]	n = 36 (19 F, 17 M), 36 teeth	18 teeth, 50% (44.4%, **55.6%**)
Zawawi et al., 2012 Saudi Arabia [16]	n = 200 (100 F, 100 M), 200 teeth	111 teeth, **55.5%** (32.4%, **67.6%**)
Shetty and Bhat, 2013 India [17]	n = 200 (75 F, 125 M), 200 teeth	108 teeth, **54%** (27.8%, **73.2%**)
Fischer et al., 2014 Germany [30]	n = 80 (46 F, 34 M), 80 teeth	42 teeth, **52.5%** (**52.4%,** 47.6%)
Yin et al., 2020 China [33]	n = 56 (43 F, 13 M), 56 teeth	39 teeth, **69.6%** (**69.2%,** 30.8%)
Peixoto et al., 2015 Portugal [19]	n = 50 (30 F, 20 M), 50 teeth	28 teeth, **56%** (50%, 50%)
Fischer et al., 2022 Germany [26]	n = 56 (36 F, 20 M), 168 teeth	99 teeth, **58.9%** (ND)
Frost et al., 2015USA [20]	n = 56 (33 F, 33 M), 306 teeth	254 teeth, **83%**’ND)
Rodrigues et al., 2022 Brazil [27]	n = 164 (116 F, 48 M), 328 teeth	170 teeth, **51.8%** (ND)
Nik-Azis et al., 2023 Malaysia [28]	n = 171 (126 F, 45 M), 171 teeth	138 teeth, **80.7%** (ND)
Transgingival probing		
Lee et al., 2018 Singapore [23]	n = 51 (27 F, 24 M), 548 teeth	256 teeth, 46.7% (ND)
Shah et al., 2015 India [18]	n = 400 (200 F, 200 M), 1200 teeth	681 teeth, **56.7%** (ND)
**Mandibular arch**		
Periodontal probe transparency test		
Fischer et al., 2022 Germany [26]	n = 56 (36 F, 20 M), 168 teeth	83 teeth, 49.4%(ND)

**Table 5:** Prevalence of thick gingiva by tooth type, according to probe transparency test or transgingival probing. F = female, M = male, ND = not determined, TG = thick gingiva. CI = central incisor, LI = lateral incisor, C = canine, PM = premolar, M = molar. Prevalences > 50% are shown in bold type.

Authors, Countries	Percentage of CI with a TG/All the CI	Percentage of IL with a TG/All the IL	Percentage of C with a TG/All the C	Percentage of PM with a TG/All the PM	Percentage of 1st M with a TG/All the 1st M
**Maxillary arch**					
Periodontal probe transparency test					
Joshi et al., 2017, India [32]	45.8%	ND	ND	ND	ND
Lee et al., 2018, Singapore [23]	10%	15%	15%	20%	**83%**
Fischer et al., 2022, Germany [26]	38.9%	ND	ND	48.3%	**94.6%**
Collins et al., 2021, Dominican Republic [25]	40.2%	ND	ND	ND	ND
Fischer et al., 2015, Germany [21]	50%	ND	ND	ND	ND
Zawawi et al., 2012, Saudi Arabia [16]	**55.5%**	ND	ND	ND	ND
Shetty and Bhat, 2013, India [17]	**54.7%**	ND	ND	ND	ND
Fischer et al., 2014, Germany [30]	**53%**	ND	ND	ND	ND
Peixoto et al., 2015, Portugal [19]	**56%**	ND	ND	ND	ND
Yin et al., 2020, China [33]	**69.6%**	ND	ND	ND	ND
Rodrigues et al., 2022, Brazil [27]	**51.8%**	ND	ND	ND	ND
Nik-Azis et al., 2023, Malaysia [28]	**80.7%**	ND	ND	ND	ND
Transgingival probing					
Lee et al., 2018, Singapore [23]	**56.2%**	31.2%	21.2%	45.9%	**86%**
**Mandibular arch**					
Periodontal probe transparency test					
Fischer et al., 2022, Germany [26]	23.2%	ND	ND	32.5%	**94.6%**
Transgingival probing					
Lee et al., 2018, Singapore [23]	7%	5%	5%	22%	**96.4%**

**Table 6:** Results of bias assessment using the Mixed Methods Appraisal Tool (Hong et al. 2018) [15]. The qualitative assessment of each study is possible by answering the five major questions with one of three possible answers: yes, no, do not know. A final score is obtained as follows: 20% is awarded for each yes answer, 0% for the other two answers. Q1: Is the sampling strategy relevant to the research question? Q2: Is the sample representative of the target population? Q3: Are the measures appropriate? Q4: Is the risk of non-response bias low? Q5: Is the statistical analysis appropriate for answering the research question?

Authors	Q 1	Q 2	Q 3	Q 4	Q 5	Scores
Zawawi et al. 2012 [16]	YES	NO	YES	NO	YES	60%
Shetty and Bhat 2013 [17]	NO	NO	NO	NO	NO	0%
Fischer et al. 2014 [30]	NO	NO	YES	NO	YES	40%
Shah et al. 2015 [18]	NO	NO	YES	NO	YES	40%
Peixoto et al. 2015 [19]	NO	NO	NO	NO	YES	20%
Frost et al. 2015 [20]	YES	NO	YES	NO	YES	40%
Fischer et al. 2015 [21]	NO	NO	YES	NO	YES	40%
Singh et al. 2016 [22]	NO	NO	YES	NO	YES	40%
Lee et al. 2018 [23]	NO	NO	YES	NO	YES	40%
Joshi et al. 2017 [32]	NO	NO	YES	NO	YES	40%
Shao et al. 2018 [31]	NO	NO	YES	NO	YES	40%
Alhajj 2020 [24]	NO	NO	YES	NO	YES	40%
Yin et al. 2020 [33]	NO	NO	YES	NO	YES	40%
Collins et al. 2021 [25]	NO	NO	YES	NO	YES	40%
Fischer et al. 2022 [26]	NO	NO	NO	NO	YES	20%
Rodrigues et al. 2022 [27]	NO	NO	YES	NO	YES	40%
Nik-Azis et al.2023 [28]	NO	NO	YES	NO	YES	40%

## Data Availability

Not applicable.
